# Psychosocial Job Strain and Sleep Quality Interaction Leading to Insufficient Recovery

**DOI:** 10.3390/ijerph10115863

**Published:** 2013-11-05

**Authors:** Leif W. Rydstedt, Jason J. Devereux

**Affiliations:** 1 Lillehammer University College (HiL), ASV, Postboks 952, 2609 Lillehammer, Norway; 2 Lloyd’s Register Consulting, University College London (UCL), 71 Fenchurch Street, London EC3M 4BS, UK; E-Mail: jason.devereux@lr.org

**Keywords:** cortisol, job strain, sleep quality

## Abstract

The purpose of the study was to assess the impact of job strain and sleep quality on the diurnal pattern of cortisol reactivity, measured by awakening and evening (10 PM) saliva cortisol. The sample consisted of 76 British white-collar workers (24 women, 52 men; mean age 45.8 years). Sleep quality and job strain were assessed in a survey distributed just before the cortisol sampling. Both input variables were dichotomized about the median and factorial ANOVA was used for the statistical analysis. Low sleep quality was significantly associated with lower morning cortisol secretion. While job strain had no main effects on the cortisol reactivity there was a significant interaction effect between the input variables on morning cortisol secretion. These findings tentatively support the hypothesis that lack of sleep for workers with high job strain may result in a flattened diurnal cortisol reactivity.

## 1. Introduction

The performance of work requires mental and physical effort resulting in short term psychological and physiological load reactions that can be experienced as physiological or emotional arousal, or in the case when individual capacity for performing work is exceeded, the experience is stress and fatigue [1,2,3]. These load reactions can result from psychosocial hazards, a term used to describe aspects of the design and management of work, and its social and organizational contexts that have the potential for causing psychological or physical harm [4]. The management of psychosocial hazards has been recognized as a major challenge to occupational health and safety, and public health [5,6]. Using broad definitions, psychosocial hazards include the job content, workload and pace, control, work schedule, organizational culture, interpersonal relationships at work, role in the organization, career development and home-work interface. 

Particular attention has although been given to the concept of psychosocial job strain. The relationship between job strain and mental [7,8] or cardiovascular health problems [9,10] has been firmly established in recent review studies. One of the most prominent conceptual models to analyze the work-related stressor-strain relationship has been the Job Strain Model [11,12]. In the original version of the model, Karasek [11] suggested job demands and control, to be the primary causal agents for work-related health outcomes. Psychosocial work demands relate to how hard and intense the job holder has to work and includes, for example, time pressure and quantitative workload. Control or decision latitude comprises two distinct but closely related components: task authority reflects the scope of the job holder’s authority to make decisions at work, while skill discretion relates to the level and variety of the skill required for the work tasks and the long-term possibilities to acquire new skills in the work role. The main effects of demands and control on work-related health outcomes have received a firm empirical support [7,13]. 

### 1.1. Job Strain and Sleep Quality

In a longitudinal study by De Lange and colleagues [14], it was found that transitions from low-strain working conditions (low demands and high control) to high-strain conditions resulted in increased sleeping problems and day time fatigue. In a Swedish longitudinal study of a mixed occupational population, a strong relationship was found between high work demands and low decision authority and, on the other hand, awakening problems measured at time 1, but the same patterns were not observed when exploring relationships between time 1 and time 2 measures [15]. 

A sleep problem has been used as an important measure in fatigue research because it has been related to, for example, an increased risk for accidents (especially in the marine sector), lowered subjective well-being as well as to mental and physical health deficits [16,17,18]. Furthermore, a large proportion of the adult population in a number of industrialized countries has been found to suffer from fatigue or various kinds of sleep problems, e.g., insomnia, disrupted sleep or low sleep quality [14,15,16,17,18,19,20,21]. It has also been found that fatigue and sleep disturbances often have a long duration [21,22]. For example, an earlier review reported the mean duration of chronic fatigue to be 10.6 years for women and 7.9 years for men [22]. Therefore, the main effects of psychosocial job strain and sleep problems as well as their interaction could have important consequences in regard to insufficiency in the recovery process during a rest period after a work shift has ended. A recent Korean survey study [23] found strong relationships between work demands and job control and outcome measures on sleep quality, disturbances and daytime dysfunction.

### 1.2. Measuring the Psycho-Physiological Response

Performance of work requires mental and physical effort which results in short term psychological and physiological load reactions. The main components of the psycho-physiological response are the sympathetic adrenal-medullary (SAM) system, regulating the secretion of the catecholamines adrenaline and noradrenaline; and the hypothalamic pituitary adrenal-cortical (HPA) system controlling cortisol secretion [24,25]. Cortisol secretion is associated with increased energy release and with suppression of the inflammatory and immune system response, which is one of its most important functions to protect the organism against its own self-defense systems [25,26]. The HPA system follows a clear circadian rhythm. Under normal conditions, the secretion of cortisol is at its lowest during the first half of a night’s sleep and, from then, the secretion gradually increases with the peak occurring the first hour after awakening and then a subsequent decrease over the course of the day to reach its nadir after midnight again [26,27]. 

The psycho-physiological cortisol response is the primary mechanism through which long term exposure to psychosocial hazards affects disease pathomechanisms. Stress-induced over-activity or disturbances of the HPA system for 6 years has been related to a 6-9 fold increase in the relative risk of cardiovascular disease (CVD) depending on prior CVD history [28], cognitive impairment [29], metabolic dys-regulation [28,29], clinical depression and upper respiratory infection following viral exposure [30]. Disturbance in the diurnal cortisol rhythm, measured as a blunted or elevated response in the morning (depending on the methodology used) and/or high levels in the evening may be an indication of insufficient recovery from long term exposure to psychosocial hazards [30,31]. 

A search of available databases did not reveal any studies that have conducted an investigation of interaction between job strain, sleep quality and its effect on psycho-physiological recovery over the course of a week. Therefore, the purpose of the present study was to analyze how job strain, sleep quality and their interactions affected awakening and evening saliva cortisol secretion measured each day over 7 consecutive days.

## 2. Experimental Section

### 2.1. Participants

The participants had previously participated in a longitudinal survey on working conditions and health outcomes [32] four years previously and volunteered to participate in the present field study. Letters were sent to 561 potential participants (white collar workers from the participating organizations that had completed the previous questionnaires), inviting them to volunteer for the study. The final sample for this study consisted of 76 British employees in white-collar occupations, 52 (68%) men and 24 (32%) women, with a mean age of 45.8 years. A comparison between the participants in the study and non-participants in the initial sampling pool revealed that there were no significant differences in age nor were there any differences in baseline job strain. The proportion of women differed although significantly (c^2^ = 8.6; degrees of freedom = 1; *p* < 0.01) between the participants in this study (32%) and the sample pool (48%). The data collection was carried out during the spring 2005 by individual visits. During the first personal meeting, each participant gave informed consent to participate in the study. 

### 2.2. Procedure

Each individual was contacted by telephone or e-mail, and meetings with a research assistant were arranged at each respondent’s place of work. At the meetings, each participant was given a paper questionnaire to complete at the beginning of the data collection week, 14 salivary collection vials, and instructions on how to use the research materials. After 7 days, the research assistant collected the materials personally.

### 2.3. Instruments

The Pittsburgh Sleep Quality Index (PSQI) was used to measure sleep quality [33]. The PSQI refers to sleep quality during the last month and consists of 19 items (each with four response alternatives), that are grouped into seven components including subjective sleep quality, sleep latency, sleep duration, habitual sleep efficiency, sleep disturbances, use of sleeping medication, and daytime dysfunction. This gives a final range from “0—very good to 21—very bad sleep quality. The PSQI has been used as an indicator of sleep quality in several previous empirical studies, where good test-retest reliability and validity have been reported [e.g., 34,35]. In the present study, the alpha coefficient was 0.63. 

The Job Content Questionnaire (JCQ), modified for the Whitehall II study [36] was used to measure job strain. Demands were measured by four items with an alpha coefficient 0.68. The items referred to working fast, working intensively, having enough time and conflicting demands. Work-related control was measured by 16 items, 10 of which assessed decision authority in the work situation (e.g., control over work tempo and methods), and six items measured skill variety (e.g., possibilities for work-related learning). The control index had had an alpha coefficient of 0.89.

Psycho-physiological response indicators as the outcome. Saliva cortisol secretion (nmol/L) was utilized as an indicator of the psycho-physiological response and was measured over seven consecutive days to include a full working week and the weekend. Individuals were contacted by telephone and/or email and meetings were arranged with a research assistant at each participant’s place of work. Two measures were taken each day: one in the morning on awakening; one at 22.00 hours. Saliva samples were collected with salivettes. The time of going to bed, the time of awakening and the exact time of every saliva sampling was recorded in a diary. Participants were instructed not to brush their teeth or drink tea, coffee or caffeinated beverages before the morning saliva sampling and were also instructed not to consume alcohol or citrus drinks 1 h before the evening saliva sampling. They stored their samples in a re-sealable plastic bag in the freezer compartment of their home refrigerator. The samples were then collected and stored at −20 degrees Celsius until transported and assayed in the laboratory at Technical University Dresden, Germany.

As recommended in the literature [37], cortisol readings of three standard deviations or more away from the group mean were considered as outlying and replaced with the individual mean for the work week readings, in accordance with standard protocol. For the outcomes measures in the study, the arithmetic means for all the readings over the full week were calculated separately for the morning and evening measures. 

### 2.4. Analytical Strategy

The input variables were dichotomized at about their medians; 39 of the participants were categorized having better sleep quality and 37 having lower sleep quality, 38 participants where categorized having low job strain and also 38 as having high job strain. Factorial ANOVA was used for the statistical analyzes.

## 3. Results

Table 1 presents the descriptive measures and inter-correlation for the variables in the study. The morning and evening saliva cortisol levels in this study were of similar magnitude to the normal values of the diurnal cortisol secretion cycle at the corresponding points of time [26], thus indicating that data set had face validity. Morning and evening cortisol secretion were moderately related. Job strain was not correlated to any of the cortisol measures.

**Table 1:** Means and inter-correlations between morning and evening saliva cortisol secretion, job strain and sleep quality. (N = 76).

	M	Sd	1	2	3	
1 Morning Cortisol	19.99	5.69				
2 Evening Cortisol	2.02	1.01	0.29*			
3 Job Strain	1.52	0.35	−0.07	−0.02		
4 PSQI	5.60	3.03	−0.25*	0.05	0.16	

* *p* < 0.05.

**Figure 1:**
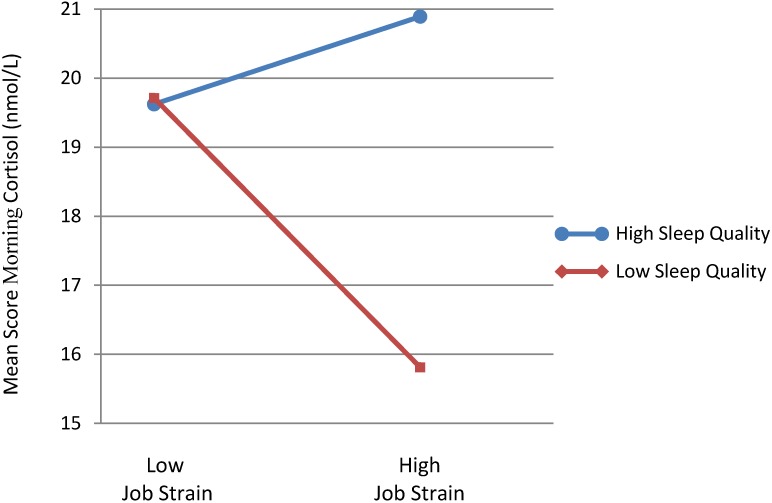
The interaction between job strain and sleep quality on morning saliva cortisol secretion.

As shown in Table 2, lower perceived sleep quality was significantly associated with reduced morning salivary cortisol (F = 4.35; *p* < 0.05), while job strain had no main effect on the morning cortisol reactivity. There was also a significant interaction effect between the independent variables (F = 4.68; *p* < 0.05)—participants with low sleep quality and high job strain had significantly reduced morning cortisol secretion over the week as shown in Figure 1 below. No significant main or interaction effects on evening cortisol secretion were found for any of the independent variables. Controlling for age and sex made the main effect of sleep quality insignificant (F = 3.08; *p* = 0.084) but the interaction sleep quality X job strain remained significant (F = 4.40; *p* = 0.039).

**Table 2:** Analysis of variance for salivary morning and evening cortisol secretion and their difference (nmol/L—for Low/High Job Strain, and Low/High Sleep Quality, N = 76). Values enclosed in parentheses represent mean square errors.

	1. Morning		2. Evening	
	df	F	df	F
Job strain (A)	1	1.21	1	0.09
Sleep quality (B)	1	4.35*	1	1.55
Interaction (A) × (B)	1	4.68*	1	1.69
Error	72	(26.1)	72	(0.93)

* *p* < 0.05.

## 4. Discussion

While job strain in itself did not affect cortisol secretion measured in the morning or the evening, it significantly interacted with perceived sleep quality to further reduce morning cortisol secretion. For participants with high job strain, there was a significant difference in the average morning cortisol reading measured over the course of a week that was dependent on whether low or high perceived sleep quality over the last month was reported. Workers reporting high job strain and lower perceived sleep quality over the last month had a lower average morning cortisol reading (16 nmol/L) compared to those reporting higher sleep quality (20 nmol/L)—about a 20% difference.

Put in another way, participants reporting high job strain and lower perceived sleep quality over the last month had the most muted psycho-physiological response measured over the course of a week. The average psycho-physiological response over the course of a week was relatively unchanged across low and high job strain groups for those reporting relatively higher sleep quality over the last month. 

Controlling for age and sex reduced the main effect of sleep quality to non-significance but the interaction between sleep quality and job strain remains significant. We consider the interaction between job strain and sleep quality as the most important finding of this study since, to the best of our knowledge, this has not been shown in any previous study. 

A recent comprehensive literature review [38] found that while most of the reviewed studies reported non-significant relationships between sleep and salivary cortisol secretion, the significant relationships were divergent. While some of them found low sleep quality to be related with increased saliva cortisol, a study by Bachaus and colleagues [39] found a significant negative relation between PSQI and morning salivary cortisol, similar to the present study. The conclusion from the review study [38] was that “the conflicting results may be at least partially due to differences in methods and underlying assumptions”.

While the PSQI referred to the sleep quality during the last month, sleep disturbances and fatigue has been shown to often have a chronic character as stated earlier [14,22,23]. Thus, the actual PSQI measure may in fact indicate more long-term sleep disturbances for many of the participants, which in turn has been suggested to be associated with hypocortisolism [31].

Relationships between job strain and sleep quality have been observed in mixed white and blue collar worker studies using self-report measures similar to our study [14,15]. Our study shows that an interaction may exist between these two factors to negatively affect psycho-physiological recovery.

It is also unclear whether the high job strain scores in this study measured at a single time point were an indication of short or long term exposure to high work demands and low job control psychosocial hazards. Although it should be mentioned that in a previous study related to this set of cortisol data [40], where an index of three consecutive job strain scores measured over a period of about 3.5 years was created, the findings failed to reveal any relationship between long term exposure to high job iso-strain (a combination of high work demands, low job control and low social support at work) and a morning saliva cortisol response. 

However, the same study found that long term exposure to job iso-strain was found to be a significant predictor of an increased evening cortisol response [39]. Those findings offered some tentative support for the hypothesis proposed by Miller and colleagues [30], which states that long term exposure to job strain may result in a flattened diurnal cortisol response pattern, characterized by decreased morning and elevated evening cortisol secretion. The present findings do also suggest that our measure of job strain when interacting with lower sleep quality may result in a blunted diurnal cortisol response. Furthermore, the recent review by Garde and colleagues has shown that studies investigating sleep duration indicate that longer sleep is related to a more dynamic cortisol secretion and that some studies have shown flat diurnal deviation associated with un-restful sleep [38,40]. Cortisol is a robust marker of overall circadian rhythm and is also a measurement of a stress response [30]. This response may indicate insufficient recovery during rest between work shifts, which is an important factor in the potential effects on safety and health at work. 

### Limitation of the Study

Since this study was based on a cross-sectional design no conclusions on causality relationships can be drawn. Furthermore, morning cortisol secretion was not assessed by the full Cortisol Awakening Response (CAR), as used in some studies, making it difficult to compare the findings from this study with other studies using CAR. Inconsistent cortisol assessment methodologies have been a major criticism in review studies on cortisol measurement [41]. On the other hand, the fact that the outcomes measures in this study were taken over a full week ought to increase their reliability. This was recognized as an important methodological issue in this study because it also investigated the difference in work day and weekend measures, reported elsewhere, which had not been done previously [40]. Measuring CAR over the full 7 days would have made the study too costly and so a decision was made to measure both a single evening and morning cortisol measure in this original explorative study over the course of an entire week. 

Job strain and perceived sleep quality were both used at the same point in time using self-report questionnaires. This may introduce a bias from common method variance. Such a bias would mean that the scores on the scales should be highly correlated. However, a high correlation was not observed suggesting that this source of study bias was minimal. Both the job demands and control scales used for formulating job strain have been previously validated in other studies against more objective methods and have been used in a number of large scale epidemiological studies.

The limited number of subjects was also a factor to consider in interpreting the results, however, the evening and morning cortisol secretion levels were in line with observations from other studies using the same saliva sample analysis method. Another shortcoming of the study was the relatively weak Chronbach’s alpha coefficients of job demands as well as of the PSQI, which to study bias.

## 5. Conclusions

The scientific evidence reviewed and collected for the International Labour Organization [6] has indicated that some multinational enterprises have observed increases in exposure to physical and psychosocial work hazards due to employee downsizing and other organizational restructuring from the most recent financial and economic crisis. The findings from our study suggest that it is prudent to monitor psychosocial work hazards, psycho-physiological recovery, subjective fatigue and health issues in occupational health and safety management systems. The findings presented in this study need to be confirmed in longitudinal studies. 
